# P-1496. Antibody Persistence of the 4CMenB Vaccine in Infants: A Systematic Literature Review

**DOI:** 10.1093/ofid/ofaf695.1680

**Published:** 2026-01-11

**Authors:** Pavo Marijic, Lucian Gaianu, Gaurav Mathur, Luca Moraschini, Thatiana Pinto, Anar Andani, Elise Kuylen, Karolina Szewczyk, Elzbieta Olewinska, Beata Smela, Helen Petousis-Harris, Stefano Castagna, Zeki Kocaata

**Affiliations:** GSK, Munich, Bayern, Germany; GSK, Munich, Bayern, Germany; GSK, Philadelphia, Pennsylvania, USA, Philadelphia, Pennsylvania; GSK, Munich, Bayern, Germany; GSK, Munich, Bayern, Germany; GSK, Munich, Bayern, Germany; GSK, Munich, Bayern, Germany; Clever-Access, Kraków, Malopolskie, Poland; Clever-Access, Kraków, Malopolskie, Poland; Clever-Access, Kraków, Malopolskie, Poland; The University of Auckland, Auckland, Auckland, New Zealand; GSK, Munich, Bayern, Germany; GSK, Munich, Bayern, Germany

## Abstract

**Background:**

Invasive meningococcal disease (IMD) incidence is highest in infants aged < 1 year, often caused by meningococcal serogroup B (MenB). The multicomponent MenB vaccine (4CMenB) contains 4 antigenic components (factor H binding protein [fHbp], *Neisseria* adhesion A [NadA], *Neisseria* heparin-binding antigen [NHBA], and outer membrane vesicles with Porin A [PorA]). This systematic literature review aimed to establish evidence-based ranges for 4CMenB antibody persistence after infant (age 0–12 months [m]) vaccination.

**Figure:**
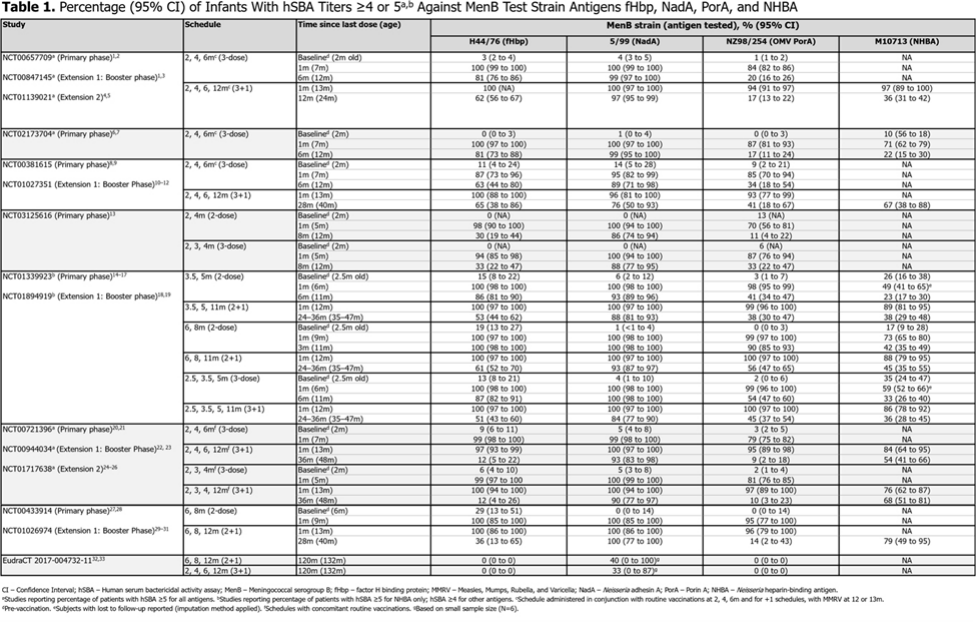

**Figure:**
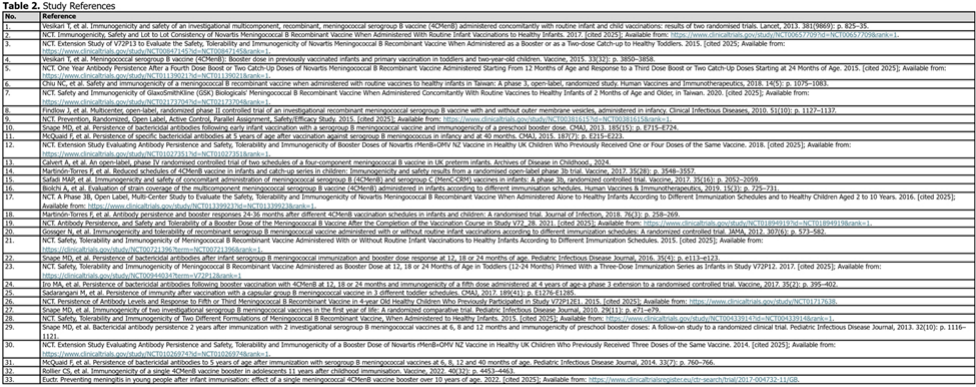

**Methods:**

MEDLINE, Embase, gray literature, and clinical trial registries were searched using prespecified criteria following PRISMA guidelines. Antibody persistence was defined as the percentage of participants with human serum bactericidal activity titers ≥ 4 or 5 against the 4CMenB antigenic components expressed in MenB strains H44/76 (fHbp), 5/99 (NadA), NZ98/254 (OMV PorA), and M10713 (NHBA).

**Results:**

Fifteen included studies (8 trials, 7 extensions; Table 1) reported antibody persistence over time and enrolled 30–3,630 participants. Variations of 2-/3-dose primary series and 3+1/2+1 booster schedules were evaluated. Antibody levels were reported from pre-vaccination up to 120m post-vaccination.

Pre-vaccination, antibody persistence was low: 0–29% (fHbp), 0–14% (NadA), 0–13% (PorA), and 10–35% (NHBA). Antibody persistence 1m after last dose was high across schedules and antigens: 87–100% (fHbp), 95–100% (NadA), 70–100% (PorA), and 49–97% (NHBA). Antibody persistence against NadA remained highest over time and against PorA decreased most rapidly. At 6–8m following last primary dose, 30–87% (fHbp), 86–100% (NadA), 11–54% (PorA), and 22–33% (NHBA) of individuals had antibody persistence. Following booster, persistence was: 62% (fHbp), 97% (NadA), 17% (PorA), and 36% (NHBA) at 12m; 12–65% (fHbp), 76–100% (NadA), 9–56% (PorA), and 36–79% (NHBA) at 24–36m; and 0% (fHbp), 33–40% (NadA), and 0% (PorA) at 120m (Table 1).

**Conclusion:**

There is strong evidence to support persistence (9–93%) of individual antibodies induced by 4CMenB (not accounting for any synergistic effect across antigens) for ≥ 36m with some evidence suggesting up to 120m (10 years) for NadA. Persistence was consistent between 2+1 and 3+1 schedules.

Funding: GSK VEO-001056

**Disclosures:**

Pavo Marijic, PhD, GSK: employee|GSK: Stocks/Bonds (Public Company) Lucian Gaianu, MSc, GSK: Employee Gaurav Mathur, MD, GSK: Employee|GSK: Stocks/Bonds (Public Company) Luca Moraschini, PhD, GSK: Employee|GSK: Stocks/Bonds (Public Company) Thatiana Pinto, PhD, GSK: employee|GSK: Stocks/Bonds (Public Company) Anar Andani, BSc, Medical director, GSK: Employee|GSK: Stocks/Bonds (Public Company) Elise Kuylen, PhD, GSK: Employee|GSK: Stocks/Bonds (Public Company) Karolina Szewczyk, MSc, GSK: Employee of Clever-Access, which was paid by GSK to conduct this study Elzbieta Olewinska, MSc, GSK: Employee of Clever-Access, which was paid by GSK to conduct this study Beata Smela, PhD, GSK: Employee of Clever-Access, which was paid by GSK to conduct this study Helen Petousis-Harris, PhD, Bexsero and gonorrhea trial (USA): Data and Safety Monitory Board Member|CDC: Funding to institution|GSK: Advisor/Consultant|GSK: Funding to institution; payment for study|Maternal pneumococcal vaccine trial (Australia): Data and Safety Monitory Board Member|New Zealand Medical Council: Advisor/Consultant|The Ministry of Health New Zealand: Funding to institution Stefano Castagna, PhD, GSK: Employee|GSK: Stocks/Bonds (Public Company) Zeki Kocaata, PhD, GSK: Employee|GSK: Stocks/Bonds (Public Company)

